# Supplementary material for: Predicting who responds to spinal manipulative therapy using a short-time frame methodology: Results from a 238-participant study
Source: PLoS One. 2020 Nov 24;15(11):e0242831. doi: 10.1371/journal.pone.0242831 (PMC7685475; doi:10.1371/journal.pone.0242831)
Supplement: Attachment — Submitted filename: Response to reviewers.docx [file pone.0242831.s002.docx]

Dear Academic Editor,

Many thanks for your valuable comments and suggestions. We have made changes to the manuscript based on these comments (please see below).

1. Why the range of age group is so wide?

Thank you for this question. We will preface our answer by underlining that the submitted manuscript is a secondary analysis of data already collected from a primary RCT. The protocol for this study can be accessed here (Trials. 2018;19(1):306) and is referenced in our manuscript. We agree though that it is very valuable for the reader to be provided with more information regarding the decisions made in designing the primary study. With respect to age range, the intent of the study was to see if specific therapeutic combinations were more effective than others. In this respect, we know of no theoretical rationale for why some age groups would respond differently than others. Therefore the age range was as wide as allowable. As such, we have now included this explanation in our manuscript to provide a better explanation for the reader of the original study design.

2. Which low back pain specify acute, sub-acute, chronic or non-specific?

As above, the original primary study was designed to assess therapeutic effects in a wide range of participants. Therefore, the original primary study did not restrict enrollment to a specific duration of low back pain. The analysis of the primary data was designed to stratify results based on pain duration which was a consideration of the original design. As such, we have now included this explanation in our manuscript to better inform the reader.

3. Why range of motion of spinal rotation is not measured?

The primary study was one that was the result of many prior studies that explored which objective measure were most responsive to the treatments under consideration. Therefore, the primary study was not designed to look for which measures might be responsive – those measures were already determined (muscle contraction viewed by ultrasound and spinal stiffness measured by indentation). Therefore, it was not appropriate to include non-responsive outcome measures, such as ROM, in the primary study. As there are many physical measures that were not included in the primary study for this reason (e.g. forward flexion), we have revised our manuscript to talk about which measures were chosen for the above reasons rather than provide a list of all the measures that were not included.

4. How sample size is calculated?

The sample size calculation was described in our protocol paper (Trials. 2018;19(1):306) and we now included this in our revised manuscript for clarification.

5. Only one technique of spinal manipulation is used explain.

As described in our above responses, the primary RCT was a culmination of many explorative studies. Not only did we identify which physical measures were responsive to the therapeutic interventions, we also showed that one specific technique was effective in providing changes to participant disability scores. As this approach has been repeated in several studies from our group (Flynn T 2002, Fritz JM 2011, Wong AY 2015, Childs JD 2004, Cleland JA 2009), it has become the standard intervention in our studies. We have now clarified this in our revised manuscript.

Journal Requirements:

1. Please ensure that your manuscript meets PLOS ONE's style requirements, including those for file naming. The PLOS ONE style templates can be found at

<https://journals.plos.org/plosone/s/file?id=wjVg/PLOSOne_formatting_sample_main_body.pdf> and

<https://journals.plos.org/plosone/s/file?id=ba62/PLOSOne_formatting_sample_title_authors_affiliations.pdf>

 The correction has been made.

2. In the ethics statement in the manuscript and in the online submission form, please provide additional information about the patient records used in your retrospective study. Specifically, please ensure that you have discussed whether all data were fully anonymized before you accessed them and/or whether the IRB or ethics committee waived the requirement for informed consent. If patients provided informed written consent to have data from their medical records used in research, please include this information.

The following statement was added to the manuscript: “All the patients’ data were fully anonymized. Permission to use anonymized data for the present study was obtained by the responsible authority, Julie M Fritz.”

3.We note that you have indicated that data from this study are available upon request. PLOS only allows data to be available upon request if there are legal or ethical restrictions on sharing data publicly. For information on unacceptable data access restrictions, please see <http://journals.plos.org/plosone/s/data-availability#loc-unacceptable-data-access-restrictions>.

In your revised cover letter, please address the following prompts:

a) If there are ethical or legal restrictions on sharing a de-identified data set, please explain them in detail (e.g., data contain potentially identifying or sensitive patient information) and who has imposed them (e.g., an ethics committee). Please also provide contact information for a data access committee, ethics committee, or other institutional body to which data requests may be sent.

b) If there are no restrictions, please upload the minimal anonymized data set necessary to replicate your study findings as either Supporting Information files or to a stable, public repository and provide us with the relevant URLs, DOIs, or accession numbers. Please see <http://www.bmj.com/content/340/bmj.c181.long> for guidelines on how to de-identify and prepare clinical data for publication. For a list of acceptable repositories, please see <http://journals.plos.org/plosone/s/data-availability#loc-recommended-repositories>.

We uploaded the anonymized data set as mentioned above.
